# A review on therapeutical potential of paeonol in atherosclerosis

**DOI:** 10.3389/fphar.2022.950337

**Published:** 2022-08-04

**Authors:** Wei Yu, Iqra Ilyas, Nasrin Aktar, Suowen Xu

**Affiliations:** ^1^ School of Materials Science and Engineering, Hefei University of Technology, Hefei, Anhui, China; ^2^ Anhui Renovo Pharmaceutical Co., Ltd., Hefei, Anhui, China; ^3^ Department of Endocrinology, The First Affiliated Hospital of USTC, Division of Life Sciences and Medicine, University of Science and Technology of China, Hefei, Anhui, China

**Keywords:** atherosclerosis, cardiovascular disease, endothelial cell, macrophage, paeonol, platelet, smooth muscle cell

## Abstract

The morbidity and mortality of atherosclerotic cardiovascular disease (ASCVD) is increasing year by year. Cortex Moutan is a traditional Chinese medicinal herb that has been widely used for thousands of years to treat a wide variety of diseases in Eastern countries due to its heat-clearing and detoxifying effects. Paeonol is a bioactive monomer extracted from Cortex Moutan, which has anti-atherosclerotic effects. In this article, we reviewed the pharmacological effects of paeonol against experimental atherosclerosis, as well as its protective effects on vascular endothelial cells, smooth muscle cells, macrophages, platelets, and other important cell types. The pleiotropic effects of paeonol in atherosclerosis suggest that it can be a promising therapeutic agent for atherosclerosis and its complications. Large-scale randomized clinical trials are warranted to elucidate whether paeonol are effective in patients with ASCVD.

## 1 Introduction

The spectrum of human diseases is gradually changing with the increasing wave of industrialization and urbanization around the world. Epidemiological studies have revealed that, with the aging process of the population, environmental pollution is increasing, alongside the acquisition of bad living habits (such as excessive intake and physical inactivity), all of which contribute to chronic diseases, especially cardiovascular disease, the leading cause of global morbidity and mortality (Clark, 2013). According to World Health Organization (WHO) studies, cardiovascular disease (CVD) is responsible for more than 40% of noncommunicable disease (NCD) mortality worldwide each year (Mendis et al., 2015).

CVD is mainly caused by atherosclerosis, a chronic lipid storage and progressive inflammatory disease of the blood vessels. The development of atherosclerosis commences with endothelial dysfunction due to endothelial cell injury, which gradually leads to the retention and accumulation of LDL and its modified form in the arterial wall. The progression of atherosclerosis leads to the narrowing and hardening of the arterial lumen due to the existence of atherosclerotic plaques (Libby, 2021). The existence of atherosclerotic plaques can be asymptomatic for decades, but sometimes the vulnerable plaques can be destabilized and susceptible to rupture. The infiltration of inflammatory cells and plaque rupture, which constrict the artery lumen, promote several life-threatening diseases in the brain and heart including ischemic stroke and myocardial infarction (Libby, 2021).

Lipid-lowering has been the cornerstone of CVD. For example, statins are the first-line therapy for the therapeutic schedule of atherosclerotic cardiovascular disease (Ferraro et al., 2020). Recently, we have witnessed the emergence of other lipid-lowering therapies, such as ATP citrate lyase (ACLY) inhibitors, etc. (Feng et al., 2020). However, due to the existence of various adverse drug reactions, such as vascular restenosis (Waksman and Iantorno 2018), drug intolerance (Nicola and Ho 2019), and rhabdomyolysis (Ben Salem et al., 2020), the benefits of intensive statin therapy need to be balanced with the potential risks they may cause. To address this dilemma, natural products have been the focus of both academics and the pharmaceutical industry. It is estimated that between 1981 and 2014, about 75% of drugs approved by the drug regulatory authorities came from natural sources or chemical semi-synthetic derivatives, mainly for the treatment of various human diseases, including cancer and cardiovascular disease (Newman and Cragg, 2016). New drug discoveries from natural products have become a new exploratory path for the therapeutics of atherosclerotic cardiovascular disease.

As a widely distributed ornamental and medicinal plant all over the world, *Paeonia suffruticosa* has aroused great research interest. The main plants with medicinal value are *P. suffruticosa* Andrew (also known as Cortex Moutan) (Adki and Kulkarni 2020). Cortex Moutan is the root bark of the peony, which was widely used in ancient China for the prevention and therapeutic treatment of various diseases, such as CVD, diabetes, arthritis, and cancer (Wang et al., 2017). It is a traditional Chinese herbal medicine that has been used for thousands of years with a good safety and efficacy profile (Wu et al., 2021). We discussed the various properties and applications of paeonol in the following section.

## 2 Paeonol

**FIGURE 1:**
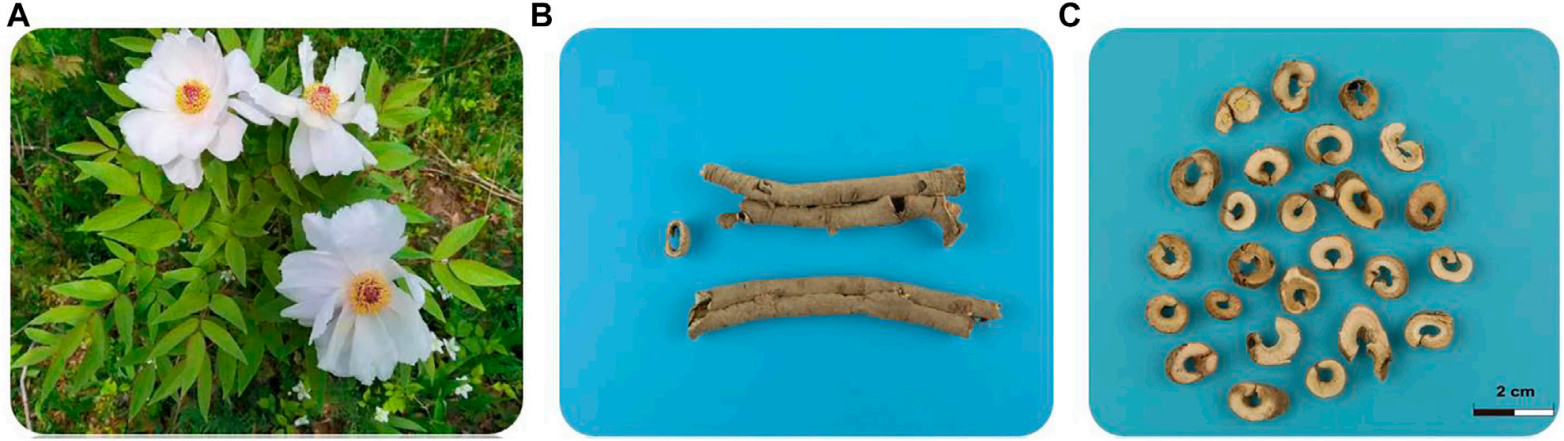
Whole plant, root bark and medicinal slices of *Paeonia suffruticosa.* **(A)** *P. suffruticosa* flowers in early May each year and lasts about a week. **(B)** Cortex Moutan, the root bark of *P. suffruticosa*, is rich in a variety of medicinal value substances, which can be used for the treatment of a variety of diseases. **(C)** Medicinal slices of Cortex Moutan.

**FIGURE 2:**
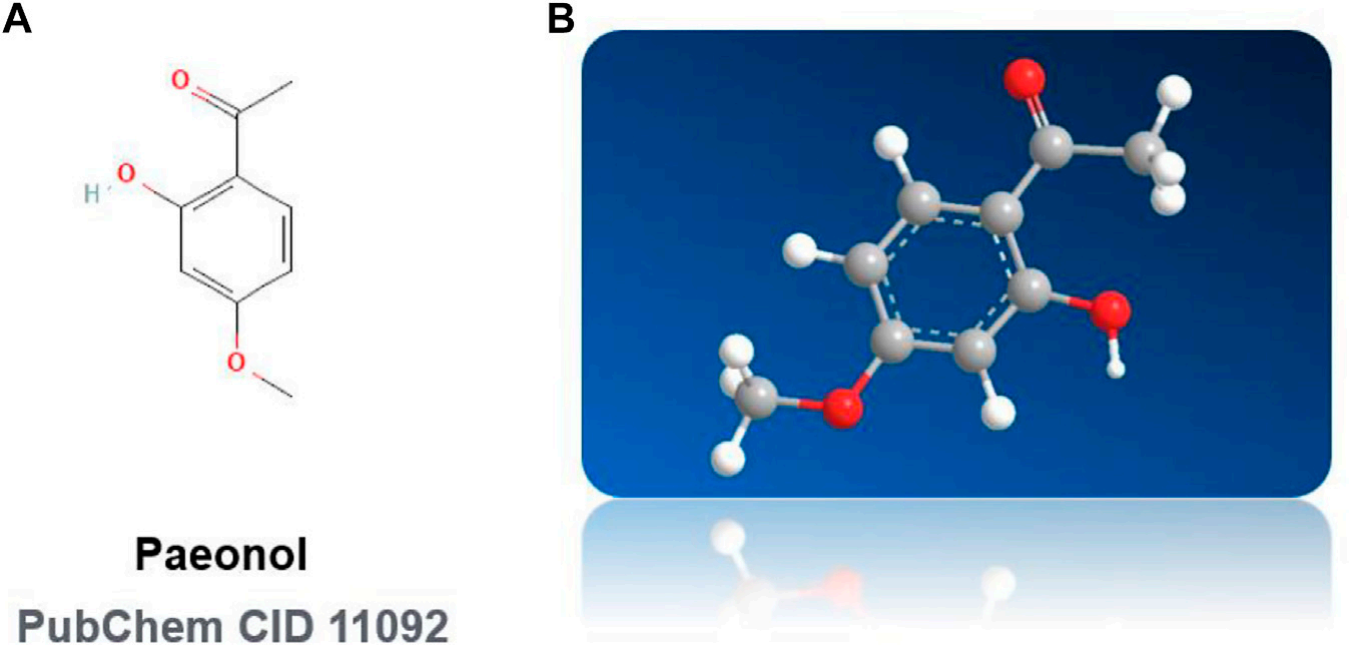
Chemical structures of paeonol. **(A)** Paeonol: 2D Chemical Structural Formula. **(B)** Paeonol: 3D Chemical Structural Formula NOTE: (Pubchem CID 11092).

Paeonol is a phenolic compound mainly isolated from the root bark and/or cortex of *P. suffruticosa* belonging to ranunculus family, the more commonly used name is Cortex Moutan, shown in Figure 1. Cortex Moutan contains a variety of bioactive components, including monoterpene glycosides, flavonoids, gallic acid derivatives, triterpenes, and particularly phenolic compounds. The primary active ingredients of Cortex Moutan include paeonol, paeoniflorin, gallic acid, and 1,2,3,4,6-penta-O-gallic acid-β-D-glucose (Wu et al., 2021). The chemical structure and 3D model of paeonol is shown in Figure 2. The relevant quality inspection standards for paeonol are specified in detail in the first part of the 2020 Edition of the Chinese Pharmacopoeia. (National Pharmacopoeia Commission, 2020), the total ash content of the cortex of medicinal materials shall not exceed 5.0% (General rule 2302), and the moisture shall not exceed 13.0% (General rule 0832 Fourth method). The pharmacopoeia stipulates that the content of paeonol (C_9_H_10_O_3_) should be determined by high performance liquid chromatography (HPLC, General rule 0512), and the content should not be less than 1.2%. At the same time, the pharmacopoeia stipulates that the ethanol extract should be determined by hot immersion method (General Rule 2201), and the content of alcohol-soluble extracts should not be less than 15%. To enhance the stability of paeonol, various derivatives of paeonol has been synthesized (Zhang et al., 2019).

### 2.1 Physiochemical properties of paeonol

Paeonol (1-(2-hydroxy-4-methoxyphenyl) ethanone) has a molecular weight of 166.17 g/mol and a melting point of 52.5°C. The oil-water partition coefficient and solubility of paeonol are different in phosphate buffer solutions with different pH values. Some data show that the oil-water partition coefficient is 461.97–981.17 μg/ml, the solubility is 284.06–598.23 μg/ml, and the effective passive permeability is 23.49 × 10^–6^ cm/s. Paeonol has an octanol/water partition coefficient of 1.98 (LogP) (Zong et al., 2017).

### 2.2 Pharmacokinetic properties of paeonol

It is well known that pharmacokinetic monitoring helps to assess the effectiveness and potential toxic effects of a drug and to explore mutual effects between different components. In recent years, some advanced detection methods have been used in the pharmacokinetic study of paeonol. One study has found that after the oral delivery method, paeonol was rapidly absorbed by the digestive system (mainly from the gastrointestinal tract) and rapidly distributed to vital organs and tissues (e.g., heart, brain, kidney, and liver) (Lu et al., 2018). Paeonol has a relatively short half-life (t½) and Tmax, rapid and complete first-pass metabolism, and low water solubility and high volatility, which contribute to its poor bioavailability in the body (Lu et al., 2018). Paeonol is mainly excreted by the urinary system, and its excretion is higher in urine than in feces (Gjertsen et al., 1988). Because paeonol is quickly removed from the human body, its safety can be ensured. In addition, studies have reported that the absorption of paeonol is a first-order process because it is independent of drug concentration and its absorption is better under acidic conditions. The rate of paeonol absorption is related to the rate of perfusion and is maximum in hypertonic solutions (Adki and Kulkarni, 2020).

### 2.3 Preparation and applications of paeonol

Based on the above-mentioned traditional and modern pharmaceutical-related research, paeonol has a lengthy history of medical applications in Northeast Asia (including China, Japan, and the Korean Peninsula). On the mainland of China, the pharmaceutical preparations of paeonol for clinical treatment date back to the 1970s (the earliest approved dosage form was an injection). At present, the dosage forms of paeonol approved by the China National Medical Products Administration (NMPA) mainly include tablets, ointments, and injections (https://www.nmpa.gov.cn/datasearch/search-result.html). According to the NMPA, the scope of indications for the instructions for paeonol drugs covers multiple fields, for instance, inflammatory or pain-related indications, including fever, headache, neuralgia, myalgia, and rheumatoid arthritis when given orally or intravenously. Topical formulations of paeonol can be used to treat skin diseases or improve skin conditions, including eczema, dermatitis, itchy skin, mosquito bites, allergic rhinitis, and colds. Furthermore, paeonol has pharmacological activities that include neuroprotective, anti-tumor, and cardiovascular protective actions along with significant anti-inflammatory properties (Zhang et al., 2019). Considering the fact that atherosclerotic cardiovascular disease is the leading cause of death worldwide, this paper focuses on evidence gleaned from experimental studies addressing paeonol’s cellular functions and mechanisms in preventing and treating atherosclerosis, including inhibitory effects on lipid accumulation, inflammatory responses, and anti-oxidation.

## 3 The effect of paeonol on experimental atherosclerosis

### 3.1 Anti-atherosclerotic effects in rabbits

Mounting evidence has suggested that the inflammatory process plays a critical role in atherosclerosis (Davis, 2005; Hansson, 2005). The inflammatory response in the blood vessel directly leads to an increase in circulating levels of inflammatory cytokines and acute-phase proteins like tumor necrosis factor (TNF)-α, interleukin (IL)-1, and C-reactive protein (CRP), which are reliable biomarkers of systemic inflammation in CVDs (Liuzzo et al., 1994; Lindahl et al., 2000) In 2009, Li et al. (2009) conducted an exploratory study to assess the anti-atherosclerotic functions of paeonol in a rabbit model of atherosclerosis by feeding rabbits a high- calorie diet for 12 weeks. The authors have shown that paeonol administration in rabbits restrains the formation and progression of atherosclerosis by inhibiting the release of inflammatory cytokines (representative inflammatory substances include: CRP, IL-1β, and TNF-α). Paeonol also significantly inhibited the nuclear translocation of nuclear factor kappa B (NF-κB, a master regulator of vascular inflammation and atherosclerosis), and inhibited lipid peroxidation, thereby significantly reduced atherosclerosis development in rabbits (Li et al., 2009).

When circulating blood rheology is abnormal, or when procoagulant substances are upregulated in atherosclerotic lesions, platelets are activated and assembled to scathing endothelium, thus initiating the coagulation cascade that leads to arterial thrombosis, vascular occlusion, and eventual life-threatening acute coronary events (Higashi et al., 2009). In addition, Shi et al. (1988) confirmed the anti-atherosclerotic effects of paeonol in rabbits fed a high-calorie diet for 6 weeks. The results showed that paeonol significantly increases the content of cyclic adenosine monophosphate (cAMP) in platelets. Paeonol improved the hemorheological parameters of rabbits by directly inhibiting platelet aggregation and blood coagulation, thereby inhibiting atherosclerosis in rabbits (Shi et al., 1988).

The above research demonstrates the therapeutic potential of paeonol for white rabbit models of AS. The main anti-atherosclerotic mechanisms of paeonol include: inhibition of platelet aggregation and blood clotting to improve blood rheological parameters; inhibits the transfer of NF-κB to the nucleus, thereby reducing the level of inflammatory factors and inhibiting lipid peroxidation.

### 3.2 Inhibition of atherosclerosis in quail

Dyslipidemia or high levels of triglyceride (TG) and total cholesterol (TC) are thought to play a critical role in the development of atherosclerosis (Chobanian, 1991). Elevated blood lipids reduce the fluidity of red blood cells, reduce their deformability, and increase the viscosity of whole blood and plasma. By feeding a high-calorie diet for 16 weeks, Dai et al. (1999) established atherosclerosis in a quail model. Oral administration of paeonol improved the lipoprotein profile and inhibited atherosclerosis in quail by significantly lowering the content of TC/TG/low-density lipoproteins (LDL)/very-low-density lipoproteins (VLDL)/apolipoprotein B100 and by upregulating the content of high-density lipoprotein (HDL) in serum. Overall, pheonol administration altered the ratios of HDL/TC, HDL2/HDL3, and apolipoprotein A1/apolipoprotein B100, thereby regulating the lipid profile, metabolism, and transport processes by altering lipoprotein composition. In quail, paeonol therapy decreased plaque area, prevented the production of aortic plaques, and thereby prevented atherosclerosis (Dai et al., 1999).

Studies of quail model of atherosclerosis have revealed the possibility that paeonol can significantly inhibit the level of “bad cholesterol” (represented by LDL cholesterol) in the serum, while increasing the level of “good cholesterol” (represented by HDL cholesterol), thereby changing the composition of lipoproteins, which in turn plays a critical role in regulating lipid metabolism and significantly inhibiting the occurrence and development of atherosclerosis.

### 3.3 Anti-atherosclerotic effect of paeonol in ApoE^−/−^ mice

Cholesterol accumulation induced by a high-fat diet is one of the common causative factors of lipid metabolism disorders and is also a high risk factor for cardiovascular disease (Sugiyama et al., 2015). ApoE^−/−^ mice are the classical animal model currently used to study the pathomechanisms and potentially new therapies of atherosclerosis. High-fat feeding combined with ApoE deficiency causes significant accumulation of lipids (mostly cholesterol, cholesterol crystals and cholesterol ester) in mice, leading to atherogenesis (Zhang B. et al., 2018). The anti-atherosclerotic effects of paeonol have been explored in ApoE^−/−^ mice in several studies.

One study by Song et al. (2018) found that paeonol can significantly reduce the plaque size in ApoE^−/−^ mice, downregulate the expression of VCAM-1 and matrix metalloproteinase-9 (MMP-9), and inhibit plaque formation. At the same time, paeonol can exert an anti-inflammatory effect and protects against oxidative stress which is involved in the initiation and development of atherosclerosis (Song et al., 2018). Another study reported that paeonol induced autophagy while activating the AMP-activated protein kinase (AMPK)/mechanistic target of rapamycin (mTOR) signaling pathway, i.e., inducing AMPK phosphorylation and reducing mTOR phosphorylation, thereby inhibiting the proliferation of VSMCs, leading to suppressive effects on atherosclerosis (Wu et al., 2018). Consistently, He et al. (2021) have confirmed that paeonol can reduce the area of aortic plaques. The authors found that paeonol can significantly reduce total cholesterol (TC) and triglyceride (TG) level in the serum and liver tissues from ApoE^−/−^ mice; Mechanistic investigations revealed that paeonol increased the content of farnesoid X receptor (FXR) antagonists (such as tauro-α-muricholic acid and tauro-β-muricholic acid, TαMCA and TβMCA), but reduced the content of FXR agonists (such as lithocholic acid, chenodeoxycholic acid, CDCA and LCA). The intestinal FXR- fibroblast growth factor 15 (FGF15) signaling pathway was also inhibited, while cholesterol 7α-hydroxylase (CYP7A1) protein expression in liver tissues was increased. The net effects of paeonol are the increase of total bile acid excretion in feces, thereby reducing cholesterol accumulation, and thus achieving its anti-atherosclerotic effect (He et al., 2021).

Liu et al. (2021) found that paeonol can induce autophagy of VSMCs *via* initiating the PI3K/Beclin-1 signaling pathway, thereby inhibiting the apoptosis of VSMCs and playing an anti-atherosclerotic role. Atherosclerosis is an epigenetic disease. It remains unknown whether paeonol inhibits atherosclerosis *via* epigenetic mechanisms. One study by Liu Y. R. et al. (2018) confirmed that paeonol inhibited the activation of STAT3 pathway in ApoE^−/−^ mice by increasing the expression of miR-223. Likewise, Liu et al. (2022) also found that paeonol inhibited the CD40/NF-κB pro-inflammatory pathway by upregulating the expression of miR-145 in ApoE^−/−^mice, thereby alleviating the inflammatory response within atherosclerotic plaques.

Overall, the above-mentioned atheroprotective effects observed in ApoE^−/−^ mice further extend the atheroprotective effects of paeonol observed in rabbit and quail atherosclerosis models. These preclinical lines of evidence in different animal models of atherosclerosis summarized in (Table 1) reinforced the confidence to develop paeonol as a promising anti-atherosclerotic drug candidate.

**TABLE 1:** Anti-atherosclerotic effects of paeonol in different animal models.

Model	Dose	Route of administration	Observed pharmacological effects	References
Rabbit	75 mg/kg, 150 mg/kg	p.o., daily for 6 weeks	TNF-α↑ IL-1β CRP NF-κB ↓ Lipid peroxidation ↓	Li et al. (2008)
	100 mg/kg	ip., daily for 6 weeks	cAMP in platelets ↑ Blood coagulation ↑ Platelet aggregation ↓	Shi et al. (1988)
Quail	300 mg/kg, 600 mg/kg	p.o., daily for 12 weeks	Tc↑ TG↓ LOL↓ VLDL↓ ApoB100↓ HDL↑ HDL/TCT↑ HDL2/HDL3↑ Apo A1/Apo B100↑	Dai et al. (1999)
ApoE^−/−^ Mice	400 mg/kg, 200 mg/kg, 100 mg/kg	p.o., daily for 6 weeks	Tc↓ TG↓ LDL↓ VCAM-1↓ MMP-9↓ IL-6↓ TNF-α↓	Song et al. (2018)
	400 mg/kg, 200 mg/kg, 100 mg/kg	p.o., daily for 6 weeks	LC3II protein ↑ AMPK/mTOR↑	Wu et al. (2018)
	200 mg/kg	p.o., daily for 20 weeks	FXR-FGF15↓ Expression of CYP7A1 ↓ FXR agonists (CDCA and LCA) ↓ FXR antagonists (TaMCA and TBMCA) ↓ Efflux of total bile acid in feces ↓	He et al. (2021)
	400 mg/kg, 200 mg/kg, 100 mg/kg	p.o., daily for 6 weeks	p62↓ Cleaved caspase-3 protein ↓ VSMCs apoptosis ↓ LC3II protein ↑ PI3K/Beclin-↑	Liu et al. (2021)
	10 mg/kg	p.o., daily for 6 weeks	miR-223↑ STAT3↑	Liu et al. (2018)
	200 mg/kg, 300 mg/kg	p.o., daily for 20 weeks	miR-145↑ CD40/NF-κ ↓ IL-1↓ IL-6↓ TNF-α↓	Liu et al. (2022)

## 4 The protective effect and mechanism of paeonol

**FIGURE 3:**
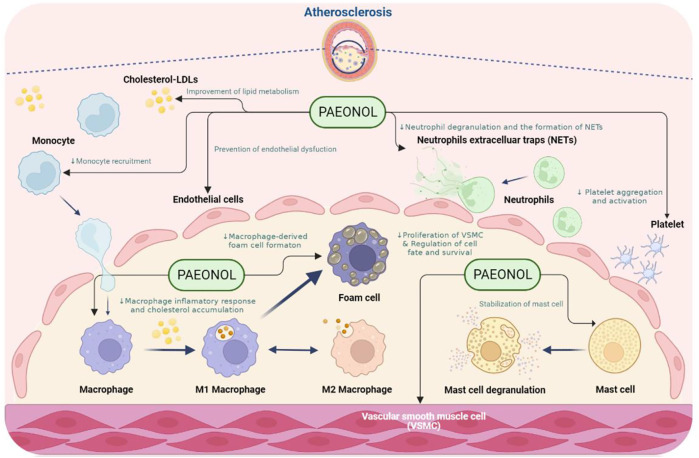
The anti-atherosclerotic effects of paeonol. NOTE: Created in BioRender.com.

Atherosclerosis is a multifaceted vascular disease involving the dysfunction of numerous cell types, such as endothelial cells, vascular smooth muscle cells, macrophages, and platelets. Dysfunction of the above-mentioned cell types leads to endothelial injury, chronic inflammation, lipid metabolism disorders, uncontrolled oxidative stress, hyperplasia, vascular smooth muscle cell proliferation and migration, and abnormal platelet activation (Weber and Noels 2011). Paeonol has protective effects on the above-mentioned cell dysfunction. The effects of paeonol on the function of each cell type implicated in atherosclerosis will be discussed in the sections below (Figure 3).

### 4.1 Paeonol and endothelial cell function

Vascular endothelial cells constitute the inner cellular lining of arteries, veins, and capillaries, which makes them the largest distribution organs in the human body (Xu et al., 2021). It is also an integral part of the anatomical and physiological functions of every tissue and organ in the body. As the outermost barrier of blood vessels, it plays a very important role in maintaining vascular homeostasis and health (Gimbrone and Garcia-Cardena 2016). When external risk factors (such as hyperlipidemia and low/oscillatory shear stress in blood flow) damage vascular endothelial cells, the function of endothelial cells is impaired, resulting in increased secretion of intercellular adhesion molecule-1 (ICAM-1) and vascular cell adhesion molecule-1 (VCAM-1) along with a battery of other adhesion molecules and/or interleukins. Furthermore, the monocytes in the blood begin to aggregate on the surface of the damaged endothelial surface and further differentiate into macrophages. These cells further secrete monocyte chemoattractant protein-1 (MCP-1), which attracts additional monocytes and leads to the production of foam cells, which is a hallmark of atherosclerosis. The release of inflammatory factors perpetuates the local inflammatory response of blood vessels and is thus involved in the initiation and progression of atherosclerosis (Habas and Shang 2018).

**FIGURE 4:**
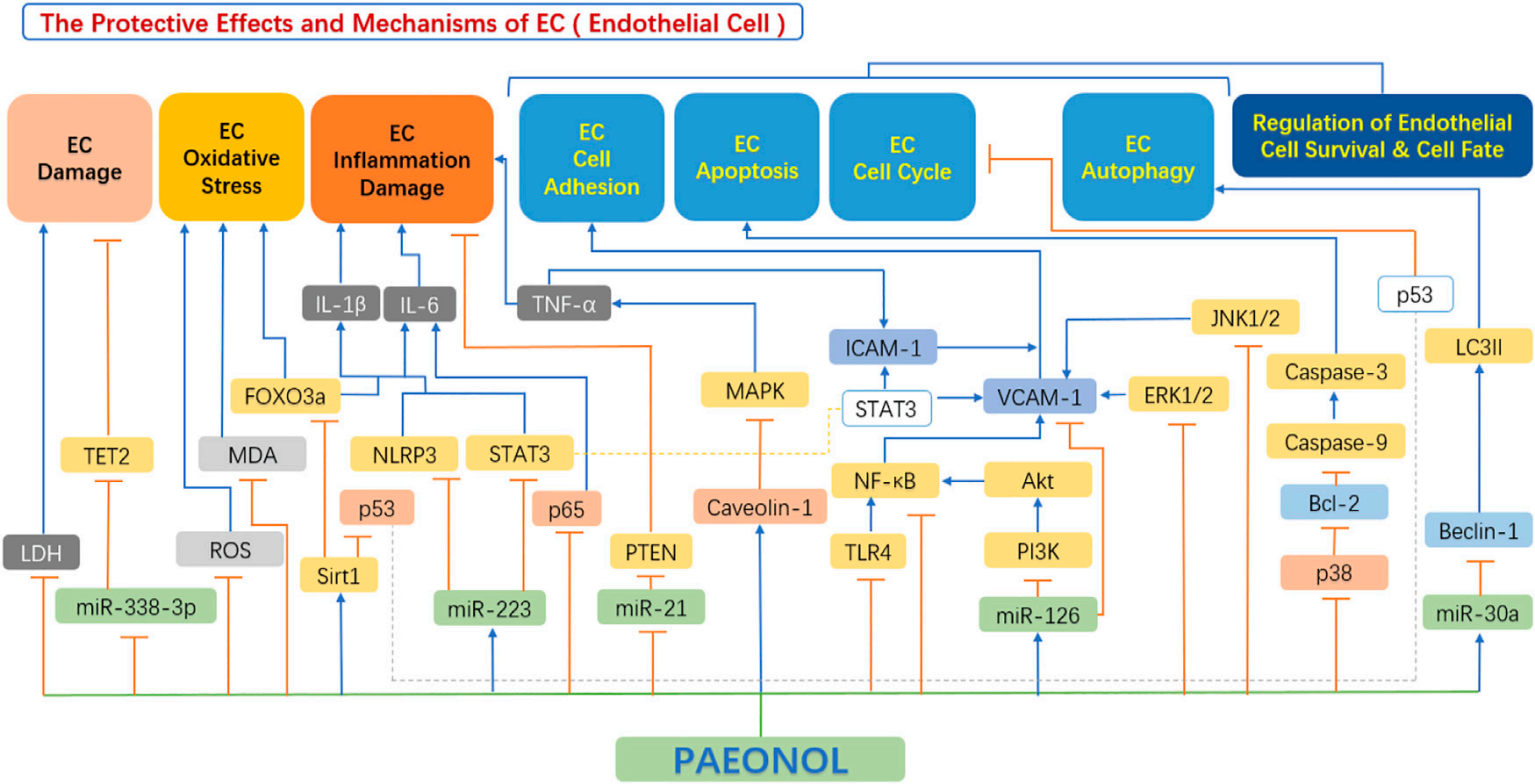
The protective effects and mechanisms of paeonol in endothelial cells. Akt, serine-threonine kinase; Bax, Bcl-2-associated X protein; Bcl-2, B-cell lymphoma-2; Beclin-1, the mammalian ortholog of yeast ATG6; ERK1/2, extracellular signal regulated kinase 1/2; FOXO3a, forkhead box transcription factor O3a; ICAM1, intercellular adhesion molecule 1; IL-1β, interleukin 1β; IL-6, interleukin 6; JNK1/2, c-Jun N-terminal kinase 1/2; LC3II, light chain 3 II; LDH, lactate dehydrogenase; MAPK, mitogen-activated protein kinase; MDA, malondialdehyde; miR-126, microRNA-126; miR-21, microRNA-21; miR-223, microRNA-223; miR-30a, microRNA-30a; miR-338-3p, microRNA-338-3p; NF-κB, the nuclear factor kB; NLRP3, NOD-like receptor 3; p38, p38 mitogen-activated protein kinase (MAPK); p53, tumor protein p53 (also known as TP53); PI3K, phosphatidylinositol-4,5-bisphosphate 3-kinase; ROS, reactive oxygen species; Sirt1, sirtuin 1; STAT3, signal transducer and activator of transcription 3; TET2, tet methyl cytosine dioxygenase 2; TLR4, toll-like receptor 4; TNF-α, tumour necrosis factor α; VCAM1, vascular cell adhesion molecule 1.

In recent years, emerging studies have demonstrated the anti-atherosclerotic effects of paeonol *via* protective effects on endothelial cell, including inhibition of endothelial cell damage, inhibition of endothelial cell oxidative stress, inhibition of the hypersecretion of inflammatory factors, improvement in endothelial cell adhesion function, and regulation of endothelial cell survival and cell fate (Figure 4).

#### 4.1.1 Inhibition of endothelial cell damage by paeonol

Vascular endothelial cell dysfunction is a primary feature of the early development of atherosclerosis (Adawi et al., 2019). Vascular endothelial cells treated with oxidized low-density lipoprotein (ox-LDL) are often used to simulate the oxidative damage of vascular endothelial cells in atherosclerosis (Zhou Z. et al., 2011). Micro-RNAs (miRNAs) are small (approximately 22 nucleotides) single strand non-coding RNAs that intercept gene expression by inhibiting transcription or accelerating the degradation of target mRNAs (Trionfini and Benigni, 2017). Studies have found an increased level of microRNA-338-3p in patients with atherosclerosis, and its overexpression accelerates ox-LDL-induced cell damage (Yin et al., 2019). Yu et al. (2020) found that paeonol could inhibit ox-LDL-induced miR-338-3p expression, by reducing Tet methylcytosine dioxygenase 2 (TET2) expression, thus regulated the development of vascular endothelial cells, and inhibited endothelial cell damage.

In addition to ox-LDL, lipopolysaccharide (LPS) is another potent pro-inflammatory stimulus that can induce the production of various inflammatory cytokines and is a widely used cellular model of inflammation-induced atherosclerosis *in vitro* (Mizia-Stec 2006). Hu et al. (2016) observed the protective effect of paeonol on LPS-induced vascular endothelial cells (VEC), co-cultured with smooth muscle cells (SMC). Endothelial cells produce more lactate dehydrogenase (LDH) after being stimulated by LPS, according to the researchers. In contrast, paeonol inhibited LPS-induced production of LDH, thereby significantly improved hyperpermeability and endothelial cell survival.

#### 4.1.2 Inhibition of oxidative stress in endothelial cells by paeonol

Oxidative stress state releases excess free radicals, and intracellular reactive oxygen species (ROS), leading to endothelial dysfunction. The generation of excessive ROS interferes with endothelial nitric oxide synthase (eNOS) function, reduces the bioavailability of the vasodilator nitric oxide (NO), and increases blood pressure (Li Q. et al., 2015). At the same time, the release of ROS further exacerbates the oxidation of LDL, leading to the formation of ox-LDL, which further damages vascular endothelial cells. In the above process, the roles of malondialdehyde (MDA) and superoxide dismutase (SOD) cannot be ignored. MDA is the outcome of lipid peroxidation *via* the attack of ROS on polyunsaturated fatty acids on biological membranes, and SOD is an important antioxidant enzyme in the body that can defend against oxidative stress (Wei et al., 2016).

The research by Bao et al. (2013) discovered that paeonol significantly inhibited ox-LDL-induced overproduction of ROS in a dose-dependent pattern, reduced endothelial cell damage caused by oxidative stress, and protected against endothelial cell dysfunction. Studies by Kim et al. (2021) also confirmed the protective effect of paeonol in endothelial cells. Tang et al. (2021) further investigated the underlying mechanism behind the protective effect of paeonol, and their research revealed that paeonol enhanced endothelial cell viability and SOD activity. At the same time, paeonol significantly lowered the levels of ROS and MDA, inhibited the expression of acetylated- fork head box O3a (FOXO3a) in high glucose and palmitic acid (HG/HP)-treated human umbilical vein endothelial cells (HUVECs) by activating the SIRT1/FOXO3a/NF-kB pathway, thereby inhibited HG/HP-induced oxidative stress in HUVECs (Tang et al., 2021).

#### 4.1.3 Inhibition of the secretion of inflammatory factors in endothelial cells

Inflammation is an integral part of the pathogenesis of multifarious cardiovascular diseases, including atherosclerosis (Soysal et al., 2020). Numerous studies have shown that paeonol has significant anti-inflammatory properties, which have been confirmed by other systems (Himaya et al., 2012; Zong et al., 2017). Acute events, such as infections and trauma, often lead to the results of acute inflammation. Many cytokines, such as TNF-α and IL-6, are released during this process, and monocytes and macrophages are activated, which activates or inhibits related signaling pathways and promotes monocyte adhesion to endothelial cells (Soysal et al., 2020). Monocytes reach the intima layer of the arterial vessel wall where they differentiate into macrophages, further exacerbate the release of inflammatory and pro-inflammatory substances such as IL-1, IL-18, and other pro-inflammatory cytokines. In the advanced phase of atherosclerosis, the accumulation of apoptotic cells and senescent cells further aggravates the state of inflammation (Libby et al., 2011). Various pro-inflammatory cytokines play central roles in the development of inflammation. Several studies have shown that paeonol restrain the formation and development of atherosclerosis by inhibiting the release of inflammatory cytokines, which is mainly concerned with the regulation of various inflammatory signaling pathways (Huang et al., 2008).

Some miRNAs play important regulatory roles in metabolic disorders and CVD (Zare et al., 2019). Shi et al. (2020) found that paeonol significantly increased the extent of miR-223 in plasma-derived exosomes of hyperlipidemia rats, inhibited the NOD-like receptor 3 (NLRP3) signaling pathway, and reduced the secretion of downstream inflammatory factors, thereby reducing the NLRP3 inflammasome-mediated endothelial cell inflammation. Also, based on the miR-223 target, Liu Y. R. et al. (2018) found that paeonol increased the expression of miR-223 in HUVECs after treatment with THP-1-derived exosomes and ingested exosomes, and inhibited STAT3 expression and activity in apolipoprotein E (ApoE^−/−^) mice. Through this pathway, the expression of STAT3 and p-STAT3 in HUVECs was reduced, followed by decreased expression of IL-1β and IL-6 (Liu Y. R. et al., 2018).

MicroRNA-21 (miR-21) plays an important role in selective expression in vascular cells, mainly regulating: cell proliferation, invasion, apoptosis, and inflammation (Kumarswamy et al., 2011). Studies have shown that, in the peripheral arterial system, if acute vascular injury occurs, miR-21 is upregulated, and the knockout of miR-21 reduces neointima formation (Ji et al., 2007). Another study conducted by Liu et al. (2014) discovered that paeonol significantly reduced the expression of miR-21 induced by ox-LDL in a dose-dependent pattern by downregulating the expression of the p38 mitogen-activated protein kinase signaling pathway (p38-MAPK), PTEN, and TNF-α release and protected endothelial cells from ox-LDL-induced damage (Liu et al., 2014). Caveolae is a specialized vesicle-like pit microstructure on the endothelial cell membrane, with caveolin as the crucial structural protein. Caveolin can negatively regulate cell signaling pathways by binding and inactivating some important molecules in several intracellular signal transduction pathways (Rodriguez-Feo et al., 2008). According to Liu et al. (2020), paeonol increased the expression of aortic caveolin-1, decreased the expression of p65 protein, inhibited the activation of the NF-B pathway, and significantly reduced serum TNF-α and IL-6 levels. Collectively, paeonol reduced inflammatory responses in vascular endothelium in a rat model of atherosclerosis.

The phosphatidylinositol 3-kinase (PI3K)/serine-threonine kinase (AKT) and NF-κB signaling pathways play a significant role in LPS-induced endothelial cell damage (Sui et al., 2008). PI3K mediates the cellular inflammatory response in endothelial cells. LPS can promote the release of cellular inflammatory factors, enhance the biological activity of PI3K, and promote the activation of AKT to regulate cell function (Chen et al., 2017). Activated NF-κB translocate to the nucleus, thereby regulating the expression of many inflammation-related specific genes such as TNF and IL-6. Studies by Wenjun Hu and Zhen Zhang found that paeonol reduced the induced protein phosphorylation in the PI3K/AKT pathway and inhibited its downstream signaling pathway, downregulated the extent of an inflammatory response, and protected endothelial cells (Hu et al., 2016). Tang et al. (2021) discovered that paeonol activated the SIRT1/FOXO3a/NF-B pathway, significantly reduced TNF-α, IL-1, and IL-6 levels, and inhibited HG/HP-induced HUVEC inflammation in a dose-dependent pattern response, thus protecting endothelial cells.

#### 4.1.4 Improve the adhesion function of endothelial cells

Cell adhesion molecules (CAMs) are membrane receptor glycoproteins that undertake very important tasks in the adhesion of cells to extracellular matrices and endothelial surfaces (Hope and Meredith, 2003). According to their structure and roles, CAMs can be divided into three main families: integrins, selectins, and the immunoglobulin superfamily. In the above classification, E-selectin, ICAM-1, and VCAM-1 play an important role in the chronic inflammatory process, and therefore have momentous relevance in CVD (Vieira et al., 2014). Selectins are binding protein molecules whose primary function is to mediate the interaction between leukocytes and endothelial cells, and subsequently, these cells form strong adhesion and migration across the endothelium. Endothelial cells express E-selectin, which is specifically and intensely stimulated by inflammatory factors like TNF-α and IL-1 and is widely expressed in the vasculature at sites of inflammation (Antoine et al., 2009).

ICAM-1 is a transmembrane glycoprotein that binds to integrins to promote the migration of leukocytes to vascular endothelial cells. With the help of ICAM-1, leukocytes adhere tightly to endothelial cells, inducing an increase in intracellular free Ca_2_+, thus affecting the contractility of myosin and the activation of p38 kinase (Lawson and Wolf, 2009). VCAM-1 is a type I transmembrane glycoprotein. Its main function is to facilitate the adhesion of leukocytes to endothelial cells. VCAM-1 is a member of the immunoglobulin superfamily and is mainly expressed in endothelial cells (Li et al., 2008). VCAM-1 is important in the development of atherosclerosis since it recruits monocyte adhesion molecules (Hwa et al., 2011). MCP-1 is the first human chemokine that has been discovered. It is produced by many cell types, including endothelial cells, fibroblasts, epithelial cells, smooth muscle cells, monocytes, and microglial cells (Singh et al., 2021). MCP-1 and its receptors (CCR2) undertake a very crucial function in the development of inflammatory responses and are vital for the recruitment of immune cells to the sites of inflammation (Deshmane et al., 2009).

MiRNA-126 (miR-126) is located in an intron of epidermal growth factor-like domain 7, and it can be selectively expressed in endothelial cells, which in turn plays an important role (Nikolic et al., 2010). Previous studies have shown that if miR-126 is knocked out, it can induce proliferation and migration of vascular endothelial cells, leading to vascular intimal injury and angiogenesis-dependent diseases (Bai et al., 2011). VCAM-1 is a specific target gene of miR-126, and silencing miR-126 resulted in increased VCAM-1 expression in TNF-stimulated VECs (Harris, 2008). Studies by Yuan et al. (2016) demonstrated that paeonol can upregulate the level of miRNA-126 in a concentration-dependent pattern, thus blocked the activation of the PI3K/Akt/NF-κB signaling pathway, and inhibited the expression of VCAM-1. Consequently, paeonol reduced the adhesion of rat monocytes to ox-LDL-injured VECs.

Under stress conditions, many cytokines and growth factors activate the mitogen-activated protein kinase (MAPK) pathway (Zhang and Liu 2002). In mammalian cells, the MAPK family consists of four members, including extracellular signal-regulated kinase (ERK), c-Jun N-terminal kinase (JNK), p38, and Big Map Kinase 1 (BMK1)/ERK5 (Pi et al., 2003; Wang et al., 2007). Previous studies have shown that cell adhesion is mainly connected with ERK1/2 and p38 (Huber et al., 2006). Wang et al. (2012) studied this signaling pathway and concluded that paeonol can inhibit the phosphorylation of ERK1/2, JNK1/2, and p38 by blocking the expression of VCAM-1, thereby prevented monocyte adhesion to ox-LDL-treated rat VECs. These preventive effects of paeonol were also verified by Pan and Dai (2009).

TNF-α is an important pro-inflammatory cytokine that causes vascular inflammation by activating the NF-B signaling pathway. Once NF-κB is dissociated from IκB-an inhibitor of NF-κB, it translocate from the cytoplasm to the nucleus and binds to a specific promoter region, which then transcribe VCAM-1 (Collins et al., 1995). Paeonol was discovered to inhibit the NF-κB signaling pathway in HUVECs by inhibiting VCAM-1 expression and preventing TNF-mediated monocyte infiltration (Kim et al., 2021). Also, based on the NF-κB signaling pathway, Yang et al. studied the role of toll-like receptor 4 (TLR4) in lipopolysaccharide-induced rat vascular endothelial cells. TLR4 is a transmembrane receptor that mediates inflammatory responses in endothelial cells. When TLR4 binds to its corresponding ligand, such as LPS, the canonical NF-κB signaling pathway is activated, leading to increased VCAM-1 protein expression (Sugiyama et al., 2008). However, paeonol can effectively inhibit the expression of TLR4, thereby inhibiting NF-κB and dependent pro-inflammatory signaling. These anti-inflammatory effects are reflected in reduced monocyte adhesion to rat endothelial cells (Li Y. et al., 2015). ICAM-1 molecule mediates the adhesion of leukocytes to activated endothelial cells by establishing strong bonds with their ligands and inducing strong leukocyte-specific β2 integrins such as lymphocyte function-associated antigen 1 (Chuang et al., 2004). Furthermore, in ApoE knockout mice, deletion of the ICAM-1 gene resulted in a marked reduction in the recruitment of monocytes to atherosclerotic lesions and was protective against atherosclerosis. These results highlight the significance of ICAM-1 in the development of atherosclerosis (Collins et al., 2000). Nizamutdinova et al. (2007) found that paeonol blocked p38, ERK, and NF-κB signaling pathways, reduced TNF-α-induced ICAM-1 expression in cultured HUVECs, and inhibited the adhesion of U937 cells to TNF-α-stimulated HUVECs. This result was also confirmed by the study by Zhou X. et al. (2011). Liu Y. R. et al. (2018) found that paeonol also inhibited the STAT3 signaling pathway, *via* not only reducing the expression of ICAM-1, but also VCAM-1 in HUVECs, thereby reduced THP-1 adhesion to HUVECs.

#### 4.1.5 Regulation of endothelial cell survival by paeonol

A large number of studies have found that paeonol can affect the fate of endothelial cells through multiple pathways, such as apoptosis, autophagy, and premature aging. Endothelial cell apoptosis leads to plaque erosion, destabilization, and thrombosis, resulting in acute symptoms in patients with coronary heart disease (Chen et al., 2005). One study has shown that autophagy in endothelial cells plays different roles at various stages of disease (Shintani and Klionsky 2004). Activation of autophagy tends to increase the survival of VECs under stressed conditions, such as oxidative damage, cellular starvation, hypoxia, and other deleterious insults (Klionsky and Codogno 2013). When tremendous accumulation of ox-LDL occurs in endothelial cells, the secretion of a large number of inflammatory factors is increased, leading to deregulated endothelial homeostasis (Fulda and Kogel 2015). However, excessive autophagy leads to dysregulation of endothelial cells, including endothelial cell damage and death (Gatica, 2015).

Cellular senescence can be defined as an irreversible state of growth arrest in proliferative normal cells with distinct phenotypes as well as dysregulated gene expression profiles and protein function (Campisi and di Fagagna 2007). In biological redox processes, high levels of oxidative stressors, such as hydrogen peroxide (H_2_O_2_) (Higashi et al., 2009), and low levels of antioxidants are imbalanced, resulting in the formation of reactive ROS in endothelial cells over time, which can promote premature endothelial cell aging (Campisi and di Fagagna 2007). Lectin-like oxidized low-density lipoprotein receptor-1 (LOX-1) is a 50 kD type II membrane glycoprotein, discovered by Sawamura et al. (1997), and belongs to the C-type lectin family. It is expressed in different cell types such as endothelial cells, macrophages, vascular smooth muscle cells, and platelets (Sawamura et al., 1997). Numerous reports have implicated LOX-1 in the pathogenesis of atherosclerosis, including endothelial cell apoptosis and dysfunction, smooth muscle cell proliferation, platelet and monocyte adhesion, and plaque rupture (Pirillo et al., 2013). Activation of LOX-1 initiates intracellular signaling (Balzan and Lubrano 2018). As a result, LOX-1 facilitates the production of ROS, which leads to the sequential phosphorylation of a series of protein kinases, such as MAPKs.

The MAPK cascade mediates the activation of multiple transcription factors, such as NF-κB, activator protein-1 (AP-1), etc., and regulates the expression of apoptosis-related proteins, such as caspase-3 (which induces apoptosis) and B cell lymphoma-2 (Bcl-2) (which prevents apoptosis). Studies have shown that paeonol can inhibit the LOX-1/p38MAPK/NF-κB pathway in HUVECs, inhibit ROS overproduction, inhibit p38MAPK phosphorylation and nuclear translocation of NF-κB, and inhibit ox-LDL-induced caspase-3 activation, thereby prevented ox-LDL-induced endothelial cell apoptosis (Bao et al., 2013). Tang et al. (2021) revealed that paeonol inhibits the expression of caspase-3, and inhibits the HG/HP-induced HUVEC apoptosis *via* regulating the SIRT1/FOXO3a/NF-κB signaling pathway. Beclin-1 is a key autophagy-promoting gene that regulates the death and survival of many cell types (Zhu et al., 2009). miR-30a has been exhibited to suppress autophagy by negatively regulating the expression of its target gene-Beclin-1 (Long et al., 2013). Li et al. (2018) found that paeonol can upregulate miRNA30a expression, thus inhibit Beclin-1 and LC3-II target genes, thereby attenuate ox-LDL-induced autophagy in VECs.

With the exception of induction of autophagy and inhibition of endothelial apoptosis, activation of Sirtuin 1 (Sirt1) also protects endothelial cells against cell death by regulating cellular senescence, cell survival, and metabolism (Inoue et al., 2007). In this regard, Jamal et al. (2014) showed that paeonol can reverse the growth-arresting effects induced by H_2_O_2_, thereby restoring endothelial cell viability. At the same time, paeonol also remarkably decreased the expression levels of p53, acetyl H3K14, and H4K16 proteins, which were upregulated by H_2_O_2_. The protective effects of paeonol are partially explained by the upregulation of Sirt1 protein and its deacetylation substrates, thereby protected endothelial cells from oxidative stress-induced premature aging (Jamal et al., 2014).

### 4.2 Paeonol and smooth muscle cell function

**FIGURE 5:**
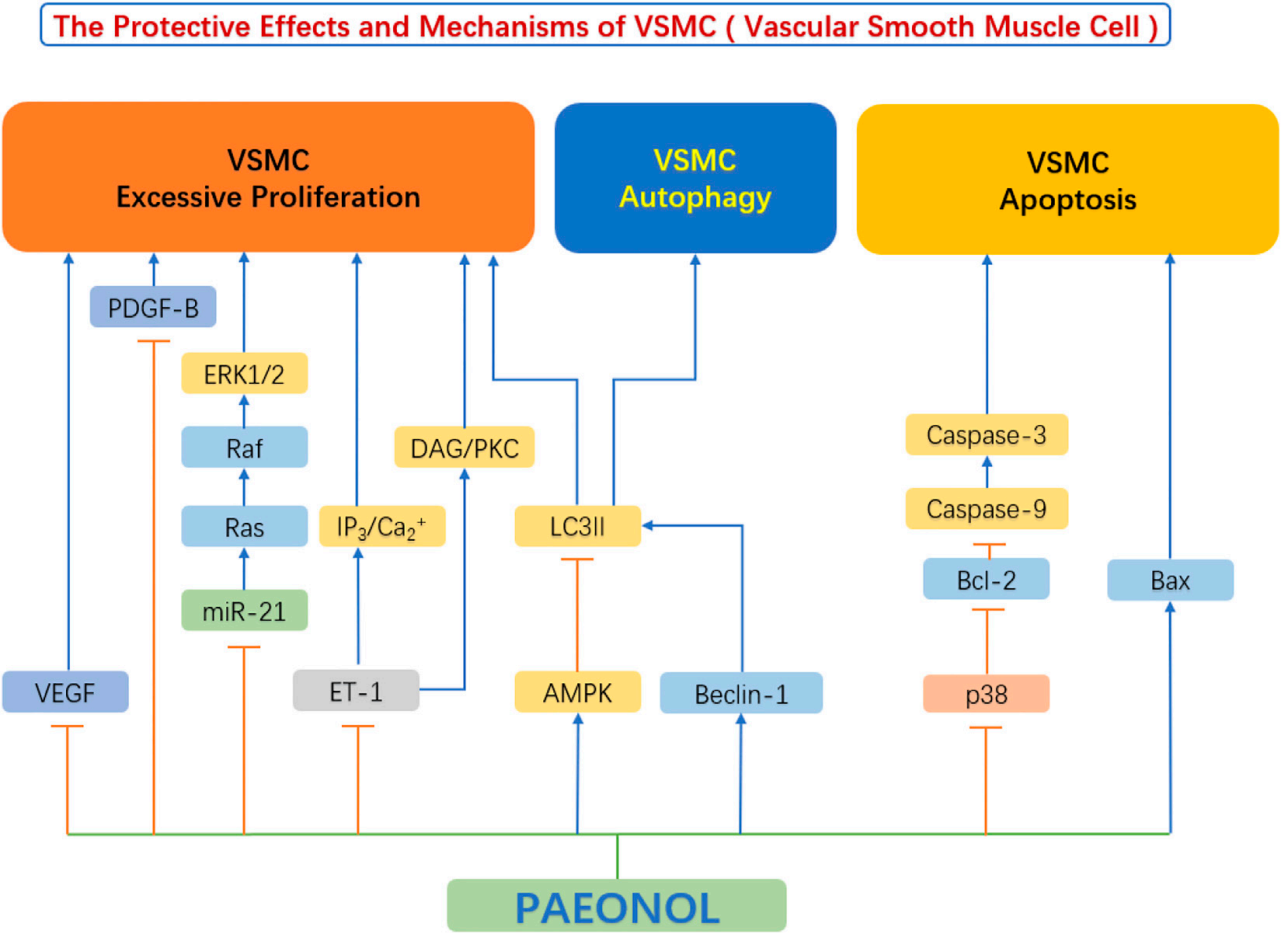
The protective effects and mechanisms of paeonol in smooth muscle cells.AMPK, AMP-activated protein kinase; Bax, Bcl-2-associated X protein; Bcl-2, B-cell lymphoma-2; Beclin-1, the mammalian ortholog of yeast ATG6; Ca, calcium; DAG, diacylglycerols; ERK1/2, extracellular signal regulated kinase 1/2; ET-1, endothelin-1; IP3, inositol 1,4,5-triphosphate; LC3II, light chain 3 II; miR-21, microRNA-21; p38, p38 mitogen-activated protein kinase (MAPK); PDGF-B, platelet derived growth factor B; PKC, protein kinase C; VEGF, vascular endothelial growth factor.

Abnormal proliferation, migration, and phenotypic switch of vascular smooth muscle cells (VSMC) are prominent manifestations of VSMC dysfunction, leading to the formation of neointima and the development of atherosclerosis (Lacolley et al., 2017). In recent years, mounting studies have shown that paeonol protects against VSMC dysfunction, by inhibiting the excessive proliferation and migration of VSMC and regulating VSMC survival (Figure 5).

#### 4.2.1 Inhibition of proliferation of vascular smooth muscle cells

Vascular endothelial cell injury and dysfunction is the leading cause of atherosclerosis development. The subsequent event following endothelial injury is the aberrant proliferation and migration of VSMC (Qin and Liu 2007). They release a large number of cytokines/chemokines/growth factors in response to endothelial injury, including Vascular Endothelial Growth Factor (VEGF), basic Fibroblast Growth Factor (bFGF), Transforming Growth Factor-β (TGF-β), Platelet Derived Growth Factor (PDGF), and others, all of which play important roles in regulating the proliferation and migration of VSMC by acting as mitogens (Laddha and Kulkarni 2019). These growth factors bind to VSMC membrane receptors and trigger the phosphorylation of downstream receptors of VEGF or PDGF-B pathways (Labrecque et al., 2005), such as ERK1/2, leading to increased VSMC proliferation and intimal thickening (Ortmann et al., 2011). Chen et al. (2014) found that paeonol can reduce the release of VEGF and PDGF-B from vascular endothelial cells. At the same time, the authors also found that paeonol can directly inhibit the Ras-Raf-ERK1/2 signaling pathway and inhibit the upregulation of Ras, Raf, and ERK phosphorylation in VSMCs (Chen et al., 2014). Further research by Wu et al. found that paeonol can also decrease the expression of endothelin-1 (ET-1), block IP3/Ca_2_+ and DAG/PKC signaling pathways, thereby inhibit vascular smooth muscle cell hyperproliferation (Wu et al., 2015).

Zhang et al. found that paeonol inhibited theERK1/2 signaling pathway and reduced the hypoxia-induced expression of cyclin A and cyclin D. By doing so, paeonol prevented the proliferation of VSMC by arresting the cell cycle at G0/G1 phase (Zhang et al., 2018). Previous studies have exhibited that AMP-dependent protein kinase (AMPK) is involved in the endothelial protective effects mediated by paeonol (Choy et al., 2016). Similarly, Wu et al. (2018) showed that the above mechanism also exists in VSMCs. Paeonol can activate the AMPK/mechanistic target of rapamycin (mTOR) signaling pathway, thereby inhibit VSMC proliferation (Wu et al., 2018).

#### 4.2.2 Regulation of smooth muscle cell fate and survival

It is well established that the apoptosis of VSMCs most often triggers plaque rupture and its sequelae (Grootaert et al., 2018). Apoptosis of VSMCs promotes multiple characteristics of vulnerable plaques, by increasing necrotic core, thinning of the fibrous cap, and infiltration of macrophages into the fibrous cap (Harrison et al., 2019). Furthermore, VSMC apoptosis leads to calcification and vascular inflammation (Dong et al., 2019).

There is evidence that high concentration of ox-LDL induce apoptosis of VSMCs and ultimately promote the formation of atherosclerosis (Ding et al., 2013). Caspase-9 plays a crucial role in the endogenous apoptotic pathway as well as caspase-3 activation (Yin et al., 2011). Meng et al. (2015) showed that paeonol can promote the upregulation of Bax in smooth muscle cells and the downregulation of Bcl-2 to induce apoptosis of vascular smooth muscle cells, thereby effectively inhibit the proliferation of VSMCs. The P38 MAPK signaling pathway plays an important role in regulating apoptosis (Gaundar and Bendall 2010), and its downstream proteins p53 and caspase-3 are closely related to apoptosis (Muller and Vousden 2014). Liu Y. et al. (2018) have shown that paeonol can inhibit the activation of the p38 MAPK signaling pathway in VSMCs, thereby reduced the level of apoptosis-related proteins, and finally inhibited the apoptosis of VSMCs. In terms of the important role of apoptosis of smooth muscle cells in driving plaque vulnerability, paeonol has the potential to stabilize vulnerable plaques (Liu Y. et al., 2018).

Unlike apoptosis, autophagy is a defense mechanism that removes unnecessary and harmful cytoplasmic components through autophagosomes. VSMC autophagy is crucial in the development of atherosclerosis (Tai et al., 2016). Autophagy plays a key protective role in maintaining cellular homeostasis and alleviating atherosclerosis progression (Liao et al., 2012). Upregulation of VSMC autophagy in the early stages of atherosclerosis helps to promote a quiescent cellular phenotype, reduces proliferation, and inhibits fibrosis (Wei et al., 2013). This process was shown to protect plaque cells from oxidative damage and maintain plaque stability (Hughes et al., 2020). In advanced stages of atherosclerosis, however, autophagy is insufficient to clear damaged mitochondria, and an intrinsic apoptotic pathway may occur (Ryter et al., 2010). In other words, autophagy abnormalities in VSMCs disrupt cellular homeostasis and cause cellular proliferation, which leads to the development or acceleration of atherosclerosis (Grootaert et al., 2015). Studies have shown that the phosphatidylinositol-3 kinase (PI3K)-Beclin-1 pathway is one of the main signaling pathways regulating autophagy (Jamuna et al., 2019). PI3Ks are thought to regulate autophagosome formation after forming a complex with Beclin-1 (Wang et al., 2020). The study by Liu et al. (2021) showed that paeonol significantly increased the level of LC3II protein in ox-LDL-induced injured VSMCs, decreased p62 and cleaved caspase-3 protein levels, and increased the number of autophagosomes of ox-LDL-injured vascular smooth muscle cells, which promotes autophagy. At the same time, the authors found that paeonol can significantly activate the PI3K/Beclin-1 signaling pathway, induce autophagy in VSMCs, and ultimately inhibit ox-LDL-induced apoptosis of vascular smooth muscle cells (Liu et al., 2021). Research by Wu et al. (2018) also showed similar results.

### 4.3 Paeonol and macrophage function

**FIGURE 6:**
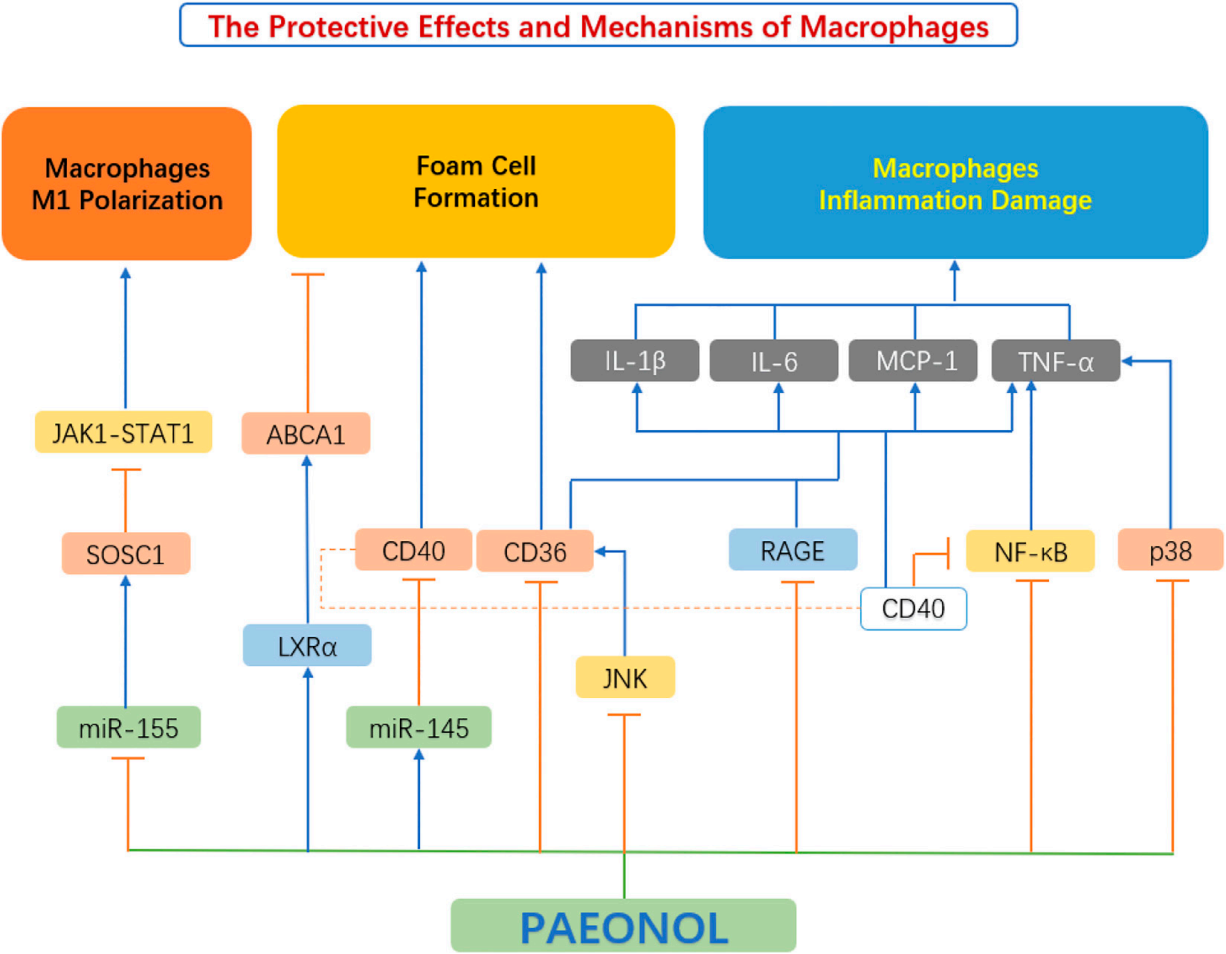
The protective effects and mechanisms of paeonol on macrophage functions. ABCA1, ATP binding cassette transporter A1; CD36, scavenger receptors; CD40, cluster of differentiation 40; IL-1β, interleukin 1β; IL-6, interleukin 6; JAK1-STAT1, Janus kinase 1-signal transducer and activator of transcription 1; JNK, c-Jun N-terminal kinase; LXRα, liver X receptor α; MCP-1, monocyte chemoattractant protein-1; miR-145, microRNA-145; miR-155, microRNA-155; NF-κB, the nuclear factor kB; P38, p38 mitogen-activated protein kinase (MAPK); RAGE, advanced glycation end products; SOCS1, suppressor of cytokine signaling 1; TNF-α, tumour necrosis factor α.

A large number of recent studies have found that paeonol has a protective effect on macrophages, which is achieved by inhibiting macrophage M1 polarization, regulating macrophage inflammation and macrophage-derived foam cell formation (Figure 6).

#### 4.3.1 Inhibition of macrophage polarization

Mounting studies have shown that the polarization of macrophages from the M1 to M2 subtype is related to the activation of Janus kinase-signal transducer and activator of the transcription (JAK-STAT) pathway. Activation of the JAK1-STAT1 pathway leads to an increase in the phosphorylation of key mediators in the JAK1-STAT1 pathway. After phosphorylation, STAT1 translocate to the nucleus, leading to the M1-type polarization of macrophages. The downstream Suppressor of Cytokine Signaling1 (SOCS1) protein is also a negative feedback regulator of this pathway. Elevation of SOCS1 inhibits the JAK1-STAT1 pathway (Feng et al., 2019; Liang et al., 2017). miR-155 is a pro-inflammatory miRNA that is highly expressed in M1-type macrophages. SOCS1 is also a target gene of miR-155. When miR-155 is elevated, the expression of SOCS1 is inhibited (Moore et al., 2013; Xu, 2016). Sun et al. stimulated RAW264.7 macrophages with LPS and IFN to establish an M1-type polarization model, then tested the effect of paeonol on macrophage polarization. It was found that paeonol reduces the expression of CD86, F4/80, IL-6, TNF-α, miR-155, and the activation of the JAK1-STAT1 pathway. Paeonol significantly downregulated the phosphorylation level of proteins involved in JAK1-STAT1 and upregulated SOCS1 protein expression. The concerted actions of paeonol reduced the polarization of RAW264.7 macrophages to M1 subtype (Sun et al., 2020).

#### 4.3.2 Regulation of macrophage-derived foam cell formation

Foam cell formation is chiefly caused by impaired cholesterol efflux or unbounded uptake of ox-LDL within macrophages (Tsai et al., 2010). Paeonol regulates lipid homeostasis by inhibiting lipid uptake and promoting cholesterol efflux in macrophages. Macrophage express a variety of scavenger receptors (SR), for example, SR-A and cluster of differentiation 36 (CD36), which are chiefly responsible for ox-LDL uptake (Zhou et al., 2013). In contrast, intracellular cholesterol efflux is primarily achieved through reverse cholesterol transporters (RCTs), mediated by ATP-binding cassette transporters a1 (ABCA1), ABCG11 and SR-BI (Saeed et al., 2012). Two of the most important factors in limiting foam cell development are scavenger receptor-mediated ox-LDL internalization and RCT-mediated cholesterol efflux. (Zhao et al., 2012).

Studies by Li X. Y. et al. (2015) showed that paeonol can reduce the expression of CD36 in RAW264.7 macrophages, thereby inhibiting the uptake of ox-LDL by macrophages, and resulting in reduced foam cell formation. Meanwhile, paeonol can also increase the expression of ABCA1 in macrophages, leading to enhanced RCT (Li X. Y. et al., 2015). Zhao et al. (2013) further investigated the mechanism by which paeonol increased the expression of ABCA1. The study found that paeonol induces ABCA1 expression *via* an LXR-dependent mechanism. By activating LXR, paeonol promoted cholesterol efflux from macrophages and reduced foam cell formation (Zhao et al., 2013).

#### 4.3.3 Inhibition of inflammatory responses in macrophages

Advanced oxidation protein products (AOPPs) are credible markers of oxidative stress and systemic inflammation, originally described in uremic subjects by Witko-Sarsat et al. (1999). AOPPs, function as a class of potential pro-inflammatory mediators in various cell types, especially macrophages, through the production of pro-inflammatory cytokines and adhesion molecules (Kishimoto et al., 2010). Studies by Ping et al. (2014) have shown that paeonol can reduce the expression level of inflammatory factors in macrophages, induced by AOPP, and promote the expression of receptors for advanced glycation end products (RAGE) and CD36 protein. Activation of the RAGE-mediated and CD36-mediated signaling pathways reduces the expression of inflammatory factors and inhibits the secretion of inflammatory factors by macrophages (Ping et al., 2014).

Jin et al. (2016) explored the effect of paeonol on the MAPK/ERK/p38 signaling pathway in macrophages and found that paeonol can dose-dependently inhibit LPS-induced phosphorylation of JNK, ERK 1/2 and p38 MAPKs, thereby attenuating the production of inflammatory factors, and improving macrophage survival.

### 4.4 Anti-platelet functions of paeonol

**FIGURE 7:**
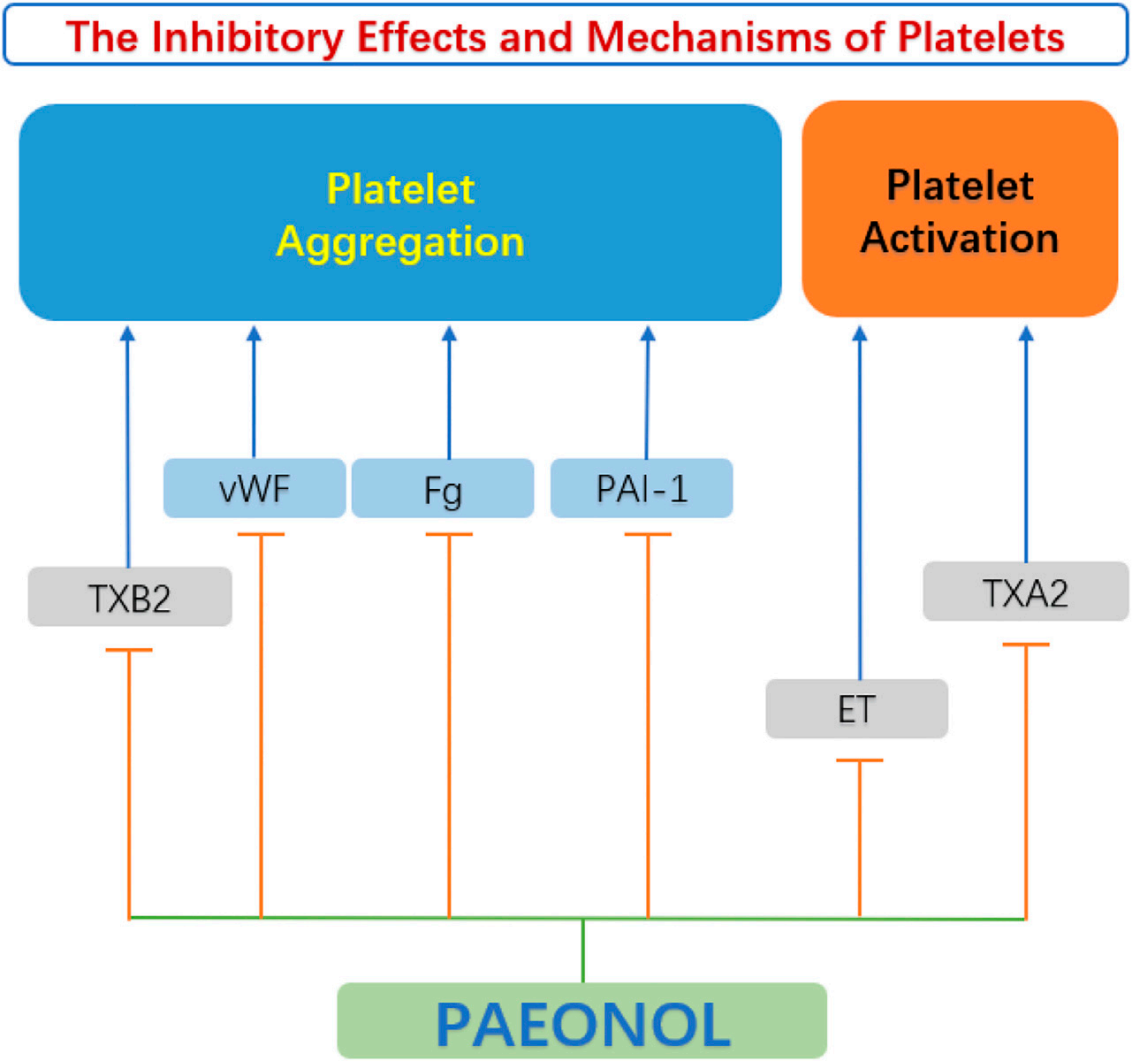
Anti-platelet function of paeonol. ET, endothelin; Fg, fibrinogen; PAI-1, plasminogen activator inhibitor 1 gene; TXA2, thromboxane A2; TXB2, thromboxane B2; vWF, von Willebrand factor.

Platelets are enucleated cells produced by megakaryocytes in the bone marrow and lungs (Lefrancais et al., 2017). Platelets are proverbially activated and recruited to impaired endothelial cells due to abnormal circulating rheology or upregulation of pro-coagulant substances in atherosclerotic lesions. The initiation of the coagulation cascade leads to arterial thrombosis and vascular occlusion. This kind of acute coronary artery disease can seriously endanger the patient’s life. (Kishimoto et al., 2010). Studies by Koo et al. (2010) elucidated that paeonol and its analogs have beneficial effects on thrombosis by directly inhibiting platelet aggregation and blood coagulation. Hirai et al. (1983) discovered that paeonol inhibited ADP-induced and collagen-induced platelet aggregation *in vitro* in a dose-dependent pattern. The mechanism of reducing platelet aggregation may be attributed to the reduction of thromboxane synthesis, along with the improvement of hemorheological parameters (Hirai et al., 1983). The anti-platelet actions of paeonol were confirmed by Bai (2015). Another anti-thrombotic mechanism of paeonol may be related to the increase of nitric oxide (NO) and prostacyclin I2 (PGI2), which are antagonists of platelet activity. While endothelin ET-1 and thromboxane A2 (TXA2) are agonists of platelet activation and aggregation, paeonol can reduce the content of ET-1 and TXA2 in the diabetic rats’ serum, and inhibit the activation and aggregation of platelets (Min et al., 2007). Therefore, paeonol is considered to be a protective agent for reducing thrombotic events (Figure 7).

### 4.5 Other protective effects and mechanisms

**FIGURE 8:**
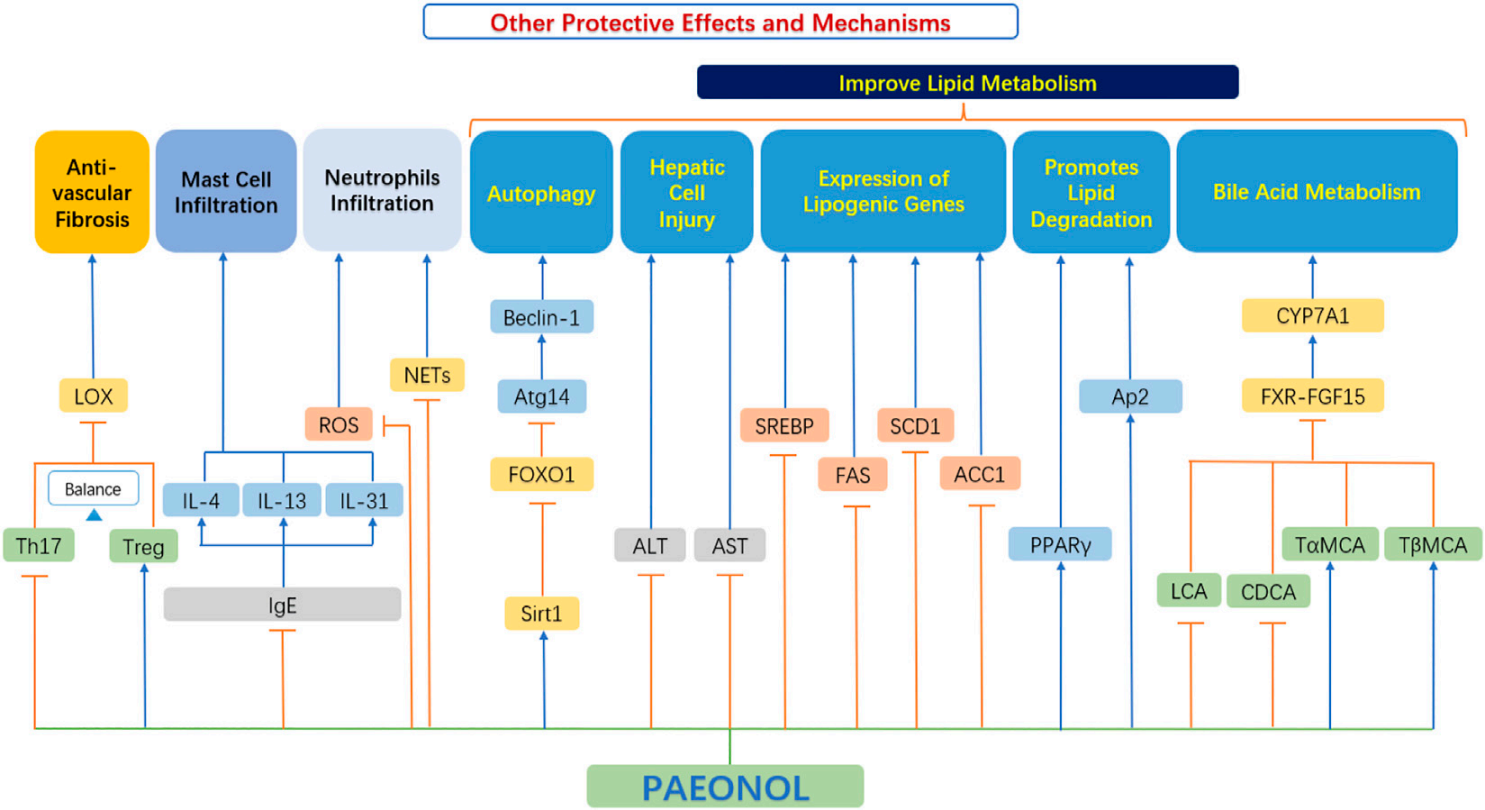
Other protective effects and mechanisms of paeonol. ACC1, acetyl-CoA carboxylase-1; ALT, alanine aminotransferase; Ap2, adipocyte protein 2; AST, aspartate aminotransferase; Atg14, autophagy related 14; Beclin-1, the mammalian ortholog of yeast ATG6; CDCA, chenodeoxycholic acid; CYP7A1, cholesterol 7α-hydroxylase; FAS, fatty acid synthase; FOXO1, forkhead box transcription factor O1; FXR, farnesoid X receptor; FGF15, fibroblast growth factor 15; IgE, immunoglobulin E; IL-4, interleukin 4; IL-13, interleukin 13; IL-31, interleukin 31; LOX, lysyl oxidase; NETs, neutrophils extracellular traps; PPAR-γ, peroxisome proliferator-activated receptor γ; ROS, reactive oxygen species; SCD1, stearoyl-CoA desaturase 1; Sirt1, sirtuin 1; SREBP, sterol regulatory element-binding protein; TαMCA, tauro-α-muricholic acid; TβMCA, tauro-β-muricholic acid; Th17, T helper type 17 cell; Treg, Regulatory T-cells.

In addition to the relevant functions mentioned above, paeonol also has many other protective effects, which play an important role in the occurrence and development of AS (Figure 8).

#### 4.5.1 Anti-fibrotic effects

Th17 cells and Treg cells both play vital roles in regulating vascular fibrosis by adjusting the expression of collagen and MMPs (Slobodin and Rimar 2017). However, a reduction in gut microbiota diversity leads to a skewed Treg/Th17 balance towards Th17 cells, which contrariwise exacerbates vascular fibrosis. Short-chain fatty acids (SCFAs) have been demonstrated to promote Treg cell differentiation and suppress inflammatory responses (Liu et al., 2020).

Shi et al. (2021) found that paeonol selectively upregulated the proportion of Treg cells and downregulated the proportion of Th17 cells in the spleen of mice. Paeonol improved the Treg/Th17 balance while indirectly downregulated the protein expression level of LOX and fibrosis-related markers (MMP-2/9 and collagen I/III), thereby reduced vascular fibrosis in a gut microbiota-dependent manner (Shi et al., 2021).

#### 4.5.2 Improvement of lipid metabolism

Dong et al. (2020) showed that paeonol activates the SIRT1-FoxO1-ATG14 signaling pathway, promotes SIRT1-FoxO1-ATG14-dependent autophagy, prevents lipid metabolism dysfunction in palmitate-induced HepG2. Hu et al. (2010) explored the beneficial effects of paeonol on alcoholic steatohepatitis in mice. Relevant studies have found that paeonol can significantly reduce the serum transaminase level in a model of alcoholic liver injury, and reduce the severity of hepatocyte injury, steatosis, and inflammatory cell infiltration. At the same time, paeonol significantly decreased the mRNA expression of hepatic lipogenesis genes and reduced hepatic lipogenesis (Hu et al., 2010). This also proves that paeonol has some potential for improving lipid metabolism.

Their studies (Li X. Y. et al., 2015) showed that paeonol can inhibit adipogenesis [inhibiting triglyceride (TG) synthesis and the expression of adipogenesis transcription factors in adipocytes], and at the same time, paeonol can promote lipid degradation in adipocytes, which in turn improves lipid metabolism.

#### 4.5.3 Stabilization of mast cells

Mast cells are primarily known for their role in host defense system and their involvement in allergies. Recently, a lot of evidence has proved that mast cells have a significant impact on cardiovascular diseases, especially on the occurrence and development of atherosclerotic plaques. Activated mast cells release growth factors, histamines and chemokines. This in turn leads to a series of cardiovascular adverse events, such as matrix degradation, apoptosis, and recruitment of inflammatory cells (Bot et al., 2015). Therefore, therapeutically, intervening in mast cell activation pathways may be more beneficial than blocking the role of individual mast cell mediator (Bot, 2021).

Kim et al. (2004) found that paeonol significantly inhibited the release of histamine by mast cells, exhibited dose-dependent inhibition of TNF-α, inhibited the production of immunoglobulin E (IgE) effectively, and downregulated the expression of IL-4 in cells, thus playing an anti-atherosclerosis role. Lee et al. (2008) also confirmed these functions. Further studies by Meng et al. (2019) found that paeonol can inhibit the level of IgE and inflammatory cytokines, decrease thymic stromal lymphopoietin, IL-4, IL-13, IL-31 and histamine, regulate T helper (Th) cell subset (Th1/Th2) ratio, and effectively inhibit mast cell infiltration, thus playing an effective protective role.

Thus, mast cell stabilization effects by paeonol appears to be a promising novel mechanism of paeonol in suppressing atherosclerosis.

#### 4.5.4 Regulation of neutrophils and neutrophils extracellular traps

Neutrophils are the main players in the pathophysiology of inflammatory diseases not only by secreting reactive oxygen species (ROS) and tissue-damaging cytotoxic enzymes, but also by releasing vital immunomodulatory cytokines and chemokines (Cross et al., 2020). There is growing evidence supporting those neutrophils are important actors in plaque progression (Döring et al., 2015). During the development of atherosclerosis, various stimulants such as platelets and chemokines are produced successively (Van Avondt et al., 2019). When neutrophils are affected, they release cytoplasmic and nuclear material, forming a network of extracellular structures that form neutrophils extracellular traps (NETs). NETs further promote inflammation and progression of atherosclerosis (Josefs et al., 2020).

The study by Chou showed that inhibition of neutrophil infiltration may be an important mechanism for paeonol to achieve anti-inflammatory effects (Chou, 2003). Fu et al. (2012) also proved the above effects. Cross et al. (2020) have recently completed a clinical trial showing that the novel drug APPA which components are apocynin (AP) and paeonol (PA) downregulates the level of ROS and neutrophil degranulation and reduces of neutrophil extracellular traps formation. In addition, APPA attenuates the expression of cytokine stimulated genes and inhibited the cell signaling induced by TNF-α and granulocyte-macrophage colony-stimulating factor (GM-CSF). All these evidence indicate a new therapeutic notion, that is, to use paeonol to intervene the progress of neutrophils and NETs (Cross et al., 2020). It should be noted that this trial is a compound preparation (APPA), so the precise effect of paeonol needs to be further verified experimentally and clinically.

## 5 Summary and outlook

Paeonol, a naturally-occurring and biologically active compound found in *P. suffruticosa*, has been demonstrated experimentally to be a promising potential therapeutic drug for the treatment of cardiovascular diseases. Paeonol has protective effects on endothelial cells, smooth muscle cells, macrophages, and platelets in the initiation and progression of atherosclerosis. These protective effects are attributed to multifactorial effects such as anti-inflammatory, anti-oxidative, anti-apoptotic, and lipid metabolism control, which are all based on separate pathways.

These pharmacological effects of paeonol indicate that it has great potential for clinical application in the prevention and treatment of atherosclerosis. We examined the drug similarity of paeonol through SwissADME website (Daina et al., 2017), and the parameters including Lipinski, Ghose, Veber, Egan, and Muegge, except for Muegge, all the other results showed that paeonol had good drug-likeness. Nevertheless, the pharmacokinetic properties of paeonol have not been well characterized. Various new dosage forms of paeonol have been developed to overcome the shortcomings of its poor solubility and instability, and to improve its oral bioavailability and drug retention in the body (Adki and Kulkarni, 2020). These efforts provided reliable technical support for paeonol in practice.

In addition, our literature review found that the atheroprotective effects of paeonol have only been addressed in rabbits, quails and ApoE knockout mice, thus lacking efficient studies in large animal models, such as rabbits and monkeys. In addition, clinical data for paeonol in patients with CVD is still insufficient. We searched the NIH clinical trial database (clinicaltrials.gov) and did not find any clinical trials using paeonol as an active ingredient for atherosclerotic diseases. Hence, large-scale randomized, placebo-controlled, and double-blinded clinical trials are urgently needed to assess the efficacy and safety of paeonol.

As summarized in this review, the molecular targets of paeonol are very complex, and the relationship (synergy and antagonism) between different targets and signaling pathways cannot be clearly explained. This urgently requires us to use systems biology methods to deeply study the specific molecular targets of paeonol for instance, the use of photoactive probes to label metformin, and then identify PEN2 as the direct target of metformin (Ma et al., 2022).

At the same time, the pathophysiology of severe acute respiratory syndrome coronavirus 2 (SARS-CoV-2) is characterized by the overproduction of inflammatory cytokines (such as IL-6 and TNF-α) leading to systemic inflammation and multiple organ dysfunction syndrome, including acute and chronic effects on the cardiovascular system (AlShahrani et al., 2021). In addition, preliminary studies indicate that vascular endothelial cells can be infected by SARS-CoV-2, and evidence of widespread endothelial injury and inflammation is found in severe patients with COVID-19 (Nagele et al., 2020). Paeonol could be a possible medication to alleviate endothelial dysfunction in COVID-19 patients due to its inhibitory effect on the expression/secretion of inflammatory cytokines.

In addition to the several major cell types mentioned above, there are many other types of cells that profoundly influence the occurrence, development, and outcome of atherosclerosis, such as immune cells (neutrophils, T/B lymphocyte cells, etc.) (Aguilar-Ballester, et al., 2020) and blood cells (red blood cells, etc.) (Blum, 2009). However, our literature review shows that there are few studies of the effect of paeonol on these cell types, and the relevant mechanisms of action and the targets and signaling pathways involved are not clear. It is well worthwhile to carry out relevant research work in the future.

Later stages of atherosclerotic progression may lead to arterial blockage, followed by myocardial hypoperfusion. This continued no-reflow may lead to severe hospitalization and death. The study of Ma et al. (2016) showed that paeonol could significantly reduce the area of myocardial infarction and no-reflow, and improve local myocardial perfusion and cardiac function, showing a potential cardiac protective effect.

In summary, paeonol exerts pleiotropic effects in different stages of atherosclerosis and may represent a promising drug candidate in pharmacotherapies of cardiovascular diseases.
